# High-resolution, large-scale laboratory measurements of a sandy beach and dynamic cobble berm revetment

**DOI:** 10.1038/s41597-021-00805-1

**Published:** 2021-01-20

**Authors:** Chris E. Blenkinsopp, Paul M. Bayle, Daniel C. Conley, Gerd Masselink, Emily Gulson, Isabel Kelly, Rafael Almar, Ian L. Turner, Tom E. Baldock, Tomas Beuzen, Robert T. McCall, Huub Rijper, Ad Reniers, Peter Troch, David Gallach-Sanchez, Alan J. Hunter, Oscar Bryan, Gwyn Hennessey, Peter Ganderton, Marion Tissier, Matthias Kudella, Stefan Schimmels

**Affiliations:** 1 Centre for Infrastructure, Geotechnics and Water Engineering, Department of Architecture and Civil Engineering, University of Bath, Bath, BA2 7AY UK; 2 School of Biological and Marine Sciences, Plymouth University, Drake Circus, PL4 8AA Plymouth, UK; 3 IRD-LEGOS, UMR5566, 18 Av. Edouard Belin, 31400 Toulouse, France; 4 Water Research Laboratory, School of Civil and Environmental Engineering, UNSW Sydney, NSW 2052 Australia; 5 School of Civil Engineering, University of Queensland, Brisbane, QLD 4072 Australia; 6 Department of Statistics, University of British Columbia, Vancouver, Canada; 7 Department of Marine and Coastal Systems, Deltares, Boussinesqweg 1, 2629 HV Delft, the Netherlands; 8 Royal Boskalis Westminster N.V., Rosmolenweg 20, 3356 LK Papendrecht, the Netherlands; 9 Faculty of Civil Engineering and Geosciences, Delft University of Technology, Stevinweg 1, 2628 CN Delft, The Netherlands; 10 Department of Civil Engineering, Ghent University, Technologiepark 60, Ghent, B-9052 Belgium; 11 DEME Group, Scheldedijk 30, 2070 Zwijndrecht, Belgium; 12 Department of Mechanical Engineering, University of Bath, Bath, BA2 7AY UK; 13 Forschungszentrum Küste (FZK), Merkurstraße 11, 30419 Hannover, Germany

**Keywords:** Physical oceanography, Natural hazards

## Abstract

High quality laboratory measurements of nearshore waves and morphology change at, or near prototype-scale are essential to support new understanding of coastal processes and enable the development and validation of predictive models. The DynaRev experiment was completed at the GWK large wave flume over 8 weeks during 2017 to investigate the response of a sandy beach to water level rise and varying wave conditions with and without a dynamic cobble berm revetment, as well as the resilience of the revetment itself. A large array of instrumentation was used throughout the experiment to capture: (1) wave transformation from intermediate water depths to the runup limit at high spatio-temporal resolution, (2) beach profile change including wave-by-wave changes in the swash zone, (3) detailed hydro and morphodynamic measurements around a developing and a translating sandbar.

## Abstract (summary)

**Table:** 

Measurement(s)	surf zone waves • swash hydrodynamics • surf zone flow velocity • morphology • sediment
Technology Type(s)	Gauge or Meter Device • Lidar • velocimetry • mechanical profiler • optical backscatter sensor • Pressure transducers
Factor Type(s)	Wave height • Wave period • beach morphology
Sample Characteristic - Environment	beach • shingle beach

Machine-accessible metadata file describing the reported data: 10.6084/m9.figshare.13194872

## Background & Summary

High quality field and numerical investigations are providing new insights into a wide variety of coastal processes and coastal protection solutions^1,2^. However, numerical modelling approaches are not yet capable of accurately reproducing all coastal hydro and morphodynamic phenomena, and the difficulties involved in capturing field data in the desired wave, tide and wind conditions mean that controlled laboratory wave flume experiments remain extremely valuable. Large-scale experiments^3,4^ are particularly valuable as they mostly avoid scaling issues, and improvements in the instrumentation and measurement techniques available mean that the quality and resolution of data continues to improve and provide new insights.

**Fig. 1:**
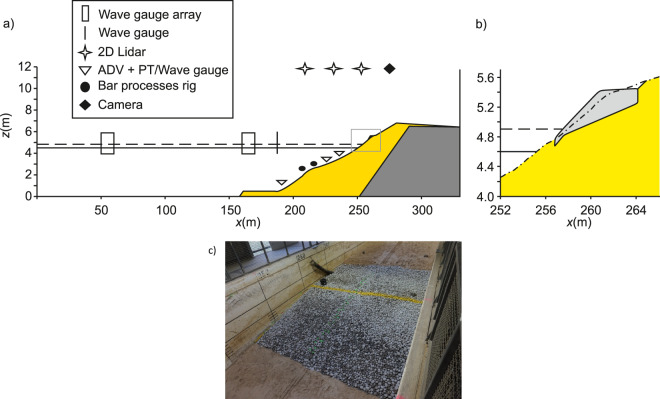
(**a**) Schematic of flume setup showing primary instrument locations (see Table 1). The yellow shaded area represents the sand volume and the dark grey shaded area is the permanent 1:6 impermeable slope. The black solid and dashed horizontal lines indicate the minimum (*z*_*wl*_ = 4.5 m) and maximum (*z*_*wl*_ = 4.9 m) water levels. (**b**) Close up of the dynamic cobble berm revetment geometry after construction corresponding to the grey box in (**a**). The minimum water level used for revetment testing (*z*_*wl*_ = 4.6 m) is shown as a solid horizontal line and the dashed line indicates the maximum water level. The light grey region indicates the constructed dynamic revetment and the dot-dashed line shows the beach profile prior to revetment construction. (**c**) Photograph of the constructed dynamic revetment on the underlying sand beach. The yellow line indicates the initial line of the revetment crest.

The DynaRev experiment was designed to investigate the response of a sand beach and the resilience of a dynamic cobble berm revetment to constant wave forcing and a rising water level at large-scale in a controlled laboratory environment through high spatio-temporal resolution morphology measurements (Fig. 1). A dynamic cobble berm revetment is a nature-based coastal protection approach which consists of a cobble ridge constructed around the high tide runup limit to artificially mimic composite beaches^5^. This commonly occurring beach type consists of a lower foreshore of sand and a backshore ridge constructed of gravel or cobbles that stabilises the upper beach and provides overtopping protection. Dynamic revetment structures contrast with static coastal defence structures as they are specifically designed to reshape under wave attack. In addition to the morphology data, high-resolution measurements of nearshore hydrodynamic processes were also collected.

DynaRev took place over a 2-month period from August to September 2017 in the 309 m long Large Wave Flume (Großer Wellenkanal, GWK), Hannover, Germany. A total of 141.6 hours of testing under wave action was completed. This testing comprised two “phases”, with each phase being split into a series of “runs” varying from 20 minutes to 3 hours in duration. The beach profile was only reset between the two phases, thus all runs had a different antecedent morphology corresponding to the beach profile at the end of the preceding run.

*Phase SB - Unmodified sand beach response to a rising water level***:** Starting with a plane 1:15 sand slope, the evolution of the beach profile was measured under constant wave forcing (*H*_*s*_ = 0.8 m, *T*_*p*_ = 6.0 s) for 20 hours. The mean water level in the flume was then raised from an initial elevation *z*_*wl*_ = 4.5 m by a total of 0.4 m in incremental steps of 0.1 m (38 hours of water level rise testing). Following the completion of the water level rise increments, the short-term response of the beach was measured at the final water level (*z*_*wl*_ = 4.9 m) for a range of different wave conditions expected to produce both erosion and accretion.

*Phase DR - Dynamic cobble berm revetment response to a rising water level:* Again starting with a manually reshaped 1:15 plane slope, a sand beach was measured as it evolved under the same constant wave conditions as used in Phase SB for 20 hours to provide a natural beach profile on which to construct the dynamic revetment. Following this, the same water level increments and test durations as for Phase SB were applied. Prior to the first water level increment, a cobble revetment was installed at the location of the sand beach berm and was designed such that its crest height was at the elevation of the *R*_*2%*_ runup level measured during Phase SB for the second water level increment to ensure significant overtopping as the water level was increased. The sand foreshore and dynamic revetment were then allowed to reshape under constant wave conditions over the remaining water level increments, with the test durations at each water level mirroring those in Phase SB (38 hours of water level rise testing). Finally, higher energy storm waves were used at the end of the final water level increment to investigate revetment resilience to higher energy conditions.

The availability to researchers of large-scale measurements of nearshore hydro and morphodynamics at the spatio-temporal resolution achieved during DynaRev is very limited. Potential uses for the datasets obtained during the DynaRev test program are wide-ranging and include: the assessment of dynamic cobble berm revetment performance^6^, the investigation of nearshore processes such as the formation and dynamics of nearshore sandbars^7^, the response of sandy coasts to a rising sea level^8^, morphology change in the swash zone^9^, wave-by-wave sediment transport rates^10^, air entrainment in breaking waves^11^ and the development of numerical models^12^.

## Methods

In this section, the experimental facility and test program are described, followed by the details of the instrumentation.

### Experimental setup and morphology

The GWK large wave flume is 309 m long, 7 m deep and 5 m wide with a combined piston-flap type wavemaker. A schematic of the experimental setup is shown in Fig. 1. All coordinates are given as the distance from the wave paddle rest position (*x* = 0 m), elevation above the horizontal flume bed (*z* = 0 m) and across-flume distance from the centreline (*y* = 0 m). The flume was filled with fresh water from the Mitteland canal which runs adjacent to the facility.

A large suite of instruments was deployed during the experiment and is detailed below. All instruments were logged by PCs connected to a local area network with a shared timeserver to ensure time-synchronisation. Table 1 lists all instruments and their locations within the flume, and the primary instrument positions are shown in Fig. 1 (noting that some instruments were moved in response to water level increases and/or evolving beach morphology).

**Table 1:** Summary of the measurement instruments deployed during the experiment including: Instrument type, measurement purpose, measurement units and primary instrument locations noting that some instruments were moved during the experiment as described in the manuscript.

Abbrev.	Instrument	Purpose (measurement units)	*x* (m)	*z* (m)
WG1	Wave gauge	Array 1: Water surface elevation in the deep flume section, *η* (m)	50	—
WG2	Wave gauge		51.9	—
WG3	Wave gauge		55.2	—
WG4	Wave gauge		60	—
WG5	Wave gauge	Array 2: Water surface elevation in the deep flume section, *η* (m)	160	—
WG6	Wave gauge		161.9	—
WG7	Wave gauge		165.2	—
WG8	Wave gauge		170	—
ADV1	Nortek Vector	Flow velocity, *u, v, w* (ms^−1^) – shoaling waves	180	2.5
ADV2	Nortek Vector	Flow velocity, *u, v, w* (ms^−1^) – surf zone	235	3.67
ADV3	Nortek Vector		242	4.22
WGADV1	Wave gauge	Water surface elevation at ADV1 location, *η* (m)	180	2.5
PTADV2	Pressure transducer	Pressure at ADV2 location, *P* (kPa)	235	3.67
PTADV3	Pressure transducer	Pressure at ADV3 location, *P* (kPa)	242	4.22
PT3	Pressure transducer	Pressure between the surf zone/ bar processes instrument rigs, *P* (kPa)	231.7	4.13
LID1	SICK LMS511 2D Lidar	High spatio-temporal resolution water surface profile, *η* (m) – surf zone	230.04	11.76
LID2	SICK LMS511 2D Lidar		242.02	11.85
LID3	SICK LMS511 2D Lidar	Swash surface profile, *η* (m), Beach/revetment *profile*, *z* (m)	254.99	11.82
CAM	Vivotek IB9381-HT high resolution camera	Swash zone imagery	Adjustable (276–280 m)	11.8
MB	Reson 7125 Multibeam	Bubble cloud, Bathymetry, *x,z* (dB)	Adjustable	Adjustable
FARO	FARO Focus 3D (Lidar)	3D topography (m)	Adjustable	Adjustable
RFID	Instrumented cobbles	Cobble movement	97 cobbles placed at 3 depths along the revetment centreline	
**Surf Zone Instrumentation** Rigs were reset to maintain constant instrument elevations above the bed at the start of every test, thus all elevations are presented in cm relative to the local bed and given the notation *h*.				
**Abbrev**.	**Instrument**	**Purpose (measurement units)**	***x*** **(m)**	***h*** **(cm)**
PT1	Pressure transducer	Pressure, *P* (kPa)	226.5	45
OBS1	Optical backscatter sensor	Suspended sediment concentration, *C* (kg/m^3^)		10
OBS2	Optical backscatter sensor			5
RPR1	Ripple Profiler	Bed profile, *z* (m)		76
EM1	Valeport Electromagnetic Current Meter	Flow velocity, *u,v* (ms^−1^)		5
EM2	Valeport Electromagnetic Current Meter			10
PT2	Pressure transducer	Pressure, *P* (kPa)	233.5	45
OBS3	Optical backscatter sensor	Suspended sediment concentration, *C* (kg/m^3^)		10
OBS4	Optical backscatter sensor			5
RPR2	Ripple Profiler	Bed profile, *z* (m)		75
EM3	Valeport Electromagnetic Current Meter	Flow velocity, *u,v* (ms^−1^)		11
EM4	Valeport Electromagnetic Current Meter			5.5

Both phases of the experiment used an initially planar sand beach with a gradient of 1:15 which was placed on top of a permanent 1:6 asphalt slope, with a minimum sand depth of 3.1 m beneath the active part of the profile (seaward of the maximum runup limit, *x* = 278 m). The beach was constructed using 1660 m^3^ of medium-coarse quartz sand (*D*_50_ = 330 µm, *D*_90_ = 650 µm and *D*_10_ = 200 µm) from the GWK facility’s material store. The sand had a density of 2650 kg/m^3^ and dry bulk density of 1680 kg/m^3^ giving a porosity of 0.37. A 25 m long layer of sand with a thickness of 0.5 m was installed in front of the slope in order to provide an additional supply of sediment. The toe of this layer was located at *x* = 161 m, the toe of the beach slope at *x* = 188.5 m and the top of the slope was at *x* = 283 m, *z* = 6.8 m (Fig. 1a).

After the first water level rise of Phase DR, a dynamic cobble berm revetment was constructed on the modified sand beach profile. The revetment was composed of 9.375 m^3^ (15 tonnes) of well sorted rounded granite cobbles with characteristics *D*_*max*_ = 90 mm, *D*_*min*_ = 50 mm, *D*_50_ = 63 mm, *D*_85_ */D*_15_ = 1.32, bulk density = 1600 kg/m^3^ and porosity = 0.41. The toe of the revetment was located at *x* = 256.8 m, *z* = 4.77 m, with a 1:6 slope leading to the crest at *x* = 260.7 m, *z* = 5.42 m. The overall height and width of the constructed revetment was 0.65 m and 7.3 m respectively. The revetment slope was selected based on guidance for recharge of shingle beaches^13^ and the crest elevation was designed to be at the elevation of the *R*_*2%*_ runup level for the second water level increment measured during Phase SB using the Lidar.

The top of the revetment extended horizontally from the crest until it intersected with the sand beach at *x* = 264.1 m, *z* = 5.42 m. Note that due to the slope of the modified sand profile approaching that of the designed revetment at the installation location, it was necessary to dig out 7.2 m^3^ of sand to enable the designed cobble volume to be placed (see Fig. 1).

### Test program

The experiment was divided into two phases corresponding to sand beach (Phase SB) and dynamic revetment (Phase DR) testing. Within each phase, the profile was monitored as it evolved under wave forcing and increasing water level. Testing within each phase was undertaken at 5 different water levels (0.1 m increments), and at each water level the experiment was divided into “runs” of increasing duration as the rate of morphological change reduced (133 runs in total). An overview of the test program is provided in Table 2 and the details of all runs are listed in the dataset associated with this paper. The initial case for both phases was a 1:15 planar sand beach with a water level *z*_*wl*_ = 4.5 m and as previously noted the beach profile was only reset between the two phases, thus all runs had a different antecedent morphology corresponding to the beach profile the end of the preceding run.

**Table 2:** Overview of the test program. The times in the program when 3D Lidar scans and RFID surveys were completed are marked with an asterisk and dagger (†) respectively in the ‘Run Durations’ column.

WL increment/Test	Duration (hr)	*H*_*s*_ (m)	*T*_*p*_ (s)	Water level *z*_*wl*_ (m)	Number of Runs	Run Durations (minutes)
**Phase SB -** ***Morphological response of a sandy beach with a rising water level***						
SB0	20	0.8	6	4.5	14	*20,20,20,30,30,60,60*,60,120,
120,120,180,180,180						
SB1	7	0.8	6	4.6	9	20,20,20,30,30,60,60,60,60,60
SB2	7	0.8	6	4.7	7	20,40,60,60,60,60,120*
SB3	7	0.8	6	4.8	7	20,40,60,60,60,60,120
SB4	17	0.8	6	4.9	11	20,40,60,60,60,60,120,120,120,180,180
**Phase SB –** ***Resilience testing at the maximum water level z***_***wl***_ = ***4.9*** ***m***						
SBE1	2	1	7	4.9	3	20,40,60
SBE2	4	1.2	8	4.9	5	20,40,60,60,60,60
SBA1	6	0.6	12	4.9	7	20,40,60,60,60,60,60*
**Phase DR –** ***Morphological response of a sandy beach with a dynamic revetment to a rising water level***						
DR0	20	0.8	6	4.5	14	*20,20,20,30,30,60,60,60,120,120,120,180,180,180*
***Dynamic revetment installation***						
DR1	7	0.8	6	4.6	9	*†20,20,20,30,30,60,60,60,120†
DR2	7	0.8	6	4.7	7	20,40,60,60,60,60,120*†
DR3	7	0.8	6	4.8	7	20,40,60,60,60,60,120*†
DR4	17	0.8	6	4.9	11	20,40,60,60,60,60,120*†,120,
120,180,180*†						
**Phase DR –** ***Resilience testing at the maximum water level z***_***wl***_ = ***4.9*** ***m***						
DRE1	2	0.9	6	4.9	3	20,40,60†
DRE2	2	1	7	4.9	4	20,20,20,60†
DRE3	1	1	8	4.9	3	20,20,20
DRR1	2	0.8	6	4.9	2	60,60
**Phase DR –** ***Resilience testing with recharged revetment at the maximum water level z***_***wl***_ = ***4.9*** ***m***						
DRN1	2	0.8	6	4.9	2	60,60^†^
DRN2	0.66	1.0	8	4.9	2	20,20
DRN3	2	0.8	6	4.9	2	60,60
DRN4	0.66	1.0	9	4.9	2	20,20
DRN5	0.33	1.2	8	4.9	1	20
DRN6	1	0.8	6	4.9	1	60

A more detailed breakdown of the test program is given in the ‘DynaRev_TestProgram.xlsx’ file provided in the dataset associated with this experiment.

#### Phase SB - Unmodified sand beach response

Starting with an initially planar slope and a water level *z*_*wl*_ = 4.5 m, the beach was first allowed to evolve naturally under constant wave forcing (*H*_*s*_ = 0.8 m, *T*_*p*_ = 6.0 s). The mean water level in the flume was raised by a total of 0.4 m in steps of 0.1 m. Measurements were undertaken for a period of 20 and 17 hours for the first (*z*_*wl*_ = 4.5 m) and final (*z*_*wl*_ = 4.9 m) water levels, and for 7 hours at the intermediate levels. This testing was divided into 63 runs with durations ranging from 20 minutes to 3 hours. Run names for this phase are given as SB < WL increment>_<Run No.>, where water level (WL) increments are numbered 0 for the initial water level of 4.5 m to 4 for *z*_*wl*_ = 4.9 m and run numbering is started from 1 for each WL increment.

Following the completion of the WL increments, “resilience testing” was completed to investigate the short-term response of the beach to a range of different wave conditions (“tests”) expected to produce both erosion and accretion. This testing was undertaken at the highest water level (*z*_*wl*_ = 4.9 m). Each test was divided into 3 to 7 runs with durations ranging from 20 to 60 minutes. These runs were labelled SBE for erosive cases and SBA for cases expected to cause accretion, numbered according to test number and then run number, *e.g*. SBE1_3 for erosive test 1, run 3.

#### Phase DR – Dynamic cobble berm revetment response

Initially, a 1:15 planar sand beach was allowed to reshape naturally under constant wave conditions (*H*_*s*_ = 0.8 m, *T*_*p*_ = 6.0 s) for 20 hours, repeating the first WL increment of Phase SB (*z*_*WL*_ = 4.5 m) to provide a natural beach profile on which to construct the dynamic cobble berm revetment. The cobble revetment was installed at the location of the sand beach berm according to the configuration given above. The revetment was designed such that it would be overtopped significantly as the water-level rose. The sand foreshore and dynamic revetment were then reshaped by waves (constant conditions; *H*_*s*_ = 0.8 m, *T*_*p*_ = 6.0 s) for the remaining water level increments, with the test durations at each water level mirroring those in Phase SB. Run names for this phase are given as DR < WL increment >_<Run No.>, where WL increments and run numbers follow those for Phase SB.

After completion of the WL increments, “resilience testing” of the revetment under varying wave conditions was undertaken at the highest water level, *z*_*wl*_ = 4.9 m. Each test was divided into 2 to 4 runs with durations ranging from 20 to 60 minutes. These runs were labelled DRE for erosive cases and DRR for cases expected to allow the revetment to recover, and numbered as per the Phase SB resilience tests.

Finally, to investigate the effect of recharging the revetment, 2.5 m^3^ of additional cobbles, corresponding to a 0.2 m thick layer were placed on the front face of the revetment. Following this recharge, the response of the revetment to a range of different high energy, erosive wave cases was measured. These runs were labelled DRN and numbered using the same notation as the resilience tests.

### Wave conditions

Wave paddle steering signals were generated according to the JONSWAP spectrum (using a peak enhancement coefficient of 3.3) specified using significant wave height, *H*_*s*_ and peak wave period *T*_*p*_. For Phases SB and DR constant wave forcing was applied, *H*_*s*_ = 0.8 m and *T*_*p*_ = 6 s. This wave condition was chosen to be mildly erosive based on experience at the BARDEX2 experiment^3^, which had a similar setup and according to criteria based on dimensionless fall velocity^14^. For each of the five water levels used, a two-hour long wave paddle signal was generated to produce an identical timeseries of waves at the wave paddle, taking water depth into account. These two-hour signals were segmented to account for the durations of the runs (20, 30, 40, 60, 120 and 180 minutes) to allow the same two-hour signal to be repeated multiple times at each WL increment with interruptions for beach profiling. Reflected waves as well as low frequency resonance were damped at the paddle using automatic reflection compensation.

For the resilience testing, erosive and accretionary wave conditions were specified primarily based on dimensionless fall velocity criteria^14–16^. The erosive cases were ordered such that the wave energy and wave runup increased with each consecutive run. Note that the wave cases used for the Phase DR resilience testing (DRE and DRR) were different to those used during Phase SB because they were modified during the experiment to investigate the observed relationship between wave period and revetment slope^6^.

### Wave measurements

The incident and reflected wave fields were measured offshore of the beach using a pair of surface-piercing resistance-capacitance wave gauge arrays, each comprising four gauges. The seaward gauges in each array were located at *x* = 50 m and *x* = 160 m, with spacings of 1.9 m, 3.3 m and 4.8 m between consecutive gauges. A further wave gauge was located at *x* = 180 m and was co-located with a Nortek Vector acoustic Doppler velocimeter (ADV) which was positioned to measure wave conditions at the toe of the sand beach slope.

**Fig. 2:**
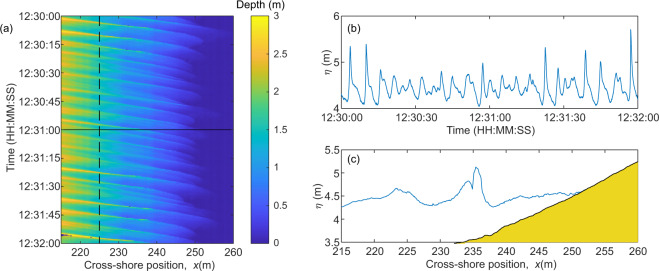
Example wave measurements. **(a)** Timestack of water depth measured by the Lidar throughout the surf and swash zones. **(b)** Timeseries of water surface elevation at *x* = 225 m as indicated by the vertical dashed line in (**a**). **(c)** Measured free- surface profile through the surf and swash zone at the time indicated by the horizontal solid line in (**a**). Note that the measurements capture the splash-up generated by a breaking wave at x = 235.5 m.

Measurements of the time-varying water surface elevation throughout the surf and swash zones were obtained using an array of three SICK LMS511 2D Lidar instruments mounted in the flume roof at an elevation, *z* = 11.8 m and at cross-shore positions *x* = 230, 242 and 255 m. The sampling rate of all three scanners was 25 Hz with an angular resolution of 0.166°. The dense spacing of the Lidars in the array ensured complete coverage of the surf and swash zones (*x* = 221.4 m to *x* = 275.8 m) throughout the experiment, with at least 12 m of overlap between the scanning regions of adjacent instruments. The use of Lidar arrays to obtain wave data throughout the surf and swash zone has been successfully demonstrated^17^. Typically, Lidar requires bubbles to be present on the water surface to ensure that the incident laser light is scattered sufficiently to obtain a valid detection. During the experiment described here, it was found that the instruments performed better than during previous field deployments^17–19^, with valid return signals even when levels of aeration were very low or in some cases, non-existent. It is thought that this was due to the presence of fine sediment in the water column which caused light to be scattered from the water surface. Example wave data obtained using the Lidar array is shown in Fig. 2.

### Morphology measurements

**Fig. 3:**
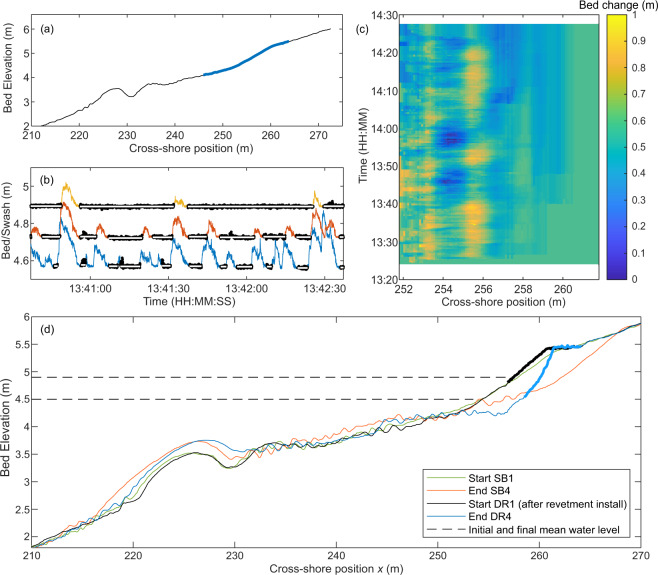
Example morphology data. **(a)** An example beach profile as measured by the mechanical profiler (black) and the swash zone profile obtained from the Lidar data (blue). **(b)** Separation of bed (black dots) and swash data at *x* = 253.8 m (blue), *x* = 255.3 m (red) and *x* = 256.8 m (orange) for an example section of data. The mean bed elevation between each swash event is shown in white. **(c)** Bed elevation change relative to the initial profile in the swash zone at the wave-by-wave timescale. **(d**) Beach profile data showing the evolution of the sand beach and dynamic revetment modified from Bayle *et al*.^6^. The revetment surface is marked with a thicker line.

The emergent and submerged beach profile, between *x* = 183 m and *x* = 270 m was measured at the end of each run using a mechanical roller attached to the overhead trolley which ran along the centre of the flume. Figure 3a shows an example profile measurement.

A Reson SeaBat 7125 multibeam echo-sounder was deployed to obtain pilot measurements of the bubble clouds generated by wave breaking^11^ and non-intrusive, regular measurements of the submerged beach profile. The echo-sounder was mounted on a vertical arm fixed to the overhead trolley of the mechanical profiler. The receiver was oriented in the vertical plane and aligned centrally along the length of the flume. A range of different cross-shore locations, depths and angles were tested to optimise data collection leading to a primary deployment position of x = 223.71, *z* = 3.8 m and an angle of 30 above the horizontal. The instrument has a 128° opening angle 0.54 beam divergence angle, operates at a frequency of 400 kHz and measurements in units of dB were collected at 1 ping per second. Note that the shallow depths and presence of bubble clouds during wave sequences make regular detection of the changing bed difficult using conventional processing methods, however new algorithms which make use of the double acoustic reflection from the water surface to the bed and back to the receiver are being developed and will be reported in future works. Due to the pilot nature of this deployment, the multiple instrument positions and orientations used, the size of the dataset and the large quantity of noisy data, the multibeam dataset is not provided in the downloadable dataset.

Wave-by-wave measurements of the changing beach face profile were obtained using the landward-most Lidar located at *x* = 255 m. Lidar detects the uppermost surface at each scan position within the swash zone – either swash surface (when submerged) or the emergent bed (between swash events). By separating the “swash” and “bed” signals within the Lidar dataset using a variance-based approach^20^ (see Fig. 3b) it is possible to obtain the beach profile landward of the swash rundown position between every swash event (Fig. 3c). The quoted error range for the Lidar is ±6 mm, however testing has demonstrated that for a stationary sand or cobble bed, this range is reduced to approximately ±0.95 mm.

Measurements of the entire three-dimensional bathymetry were obtained at irregular intervals when the flume was drained using a FARO Focus 3D terrestrial laser scanner. A total of 11 surveys of this type were completed throughout the duration of the experiment.

### Surf Zone/Sandbar measurements

Two measurement rigs were installed immediately landward and seaward of the predicted sandbar location and each housed an array of instrumentation designed to measure hydrodynamics, sediment transport and morphological change during bar formation and migration. The main instrument mounting bars for these rigs were located at *x* = 226.5 and 233.5 m. Each of the measurement rigs was fixed to the walls on a mechanism such that they could be lifted and lowered manually to the bed after each run to ensure that all instruments remained a constant height above the evolving bed (see Table 1).

Each rig was equipped with the following instruments which were sampled at 8 Hz: 2 optical backscatter sensors (OBS) mounted at 5 and 10 cm from the bed, two electromagnetic current meters (EMCM) at elevations of 5 and 10 cm above the bed and a pressure transducer (PT) mounted 45 cm above the bed. The error ranges of the EMCMs and PTs are approximately ±0.015 ms^−1^ and ±0.6 Pa respectively. Finally, a ripple profile scanner (RPS) was mounted 75 cm above the bed to obtain local bed profile measurements along a 0.9 m transect. The RPS on each rig was sampled alternately for one minute to avoid crosstalk between instruments.

In addition to the two rigs, two Nortek ADVs were located at *x* = 235 and 242 m, maintained at a height 15 cm above the bed and sampled at 25 Hz. Each ADV was co-located with a pressure transducer and an additional standalone pressure transducer was installed at *x* = 231.7 m, *z* = 4.13 m. The error range for the ADVs for the velocities measured is approximately ±0.01 ms^−1^.

**Fig. 4:**
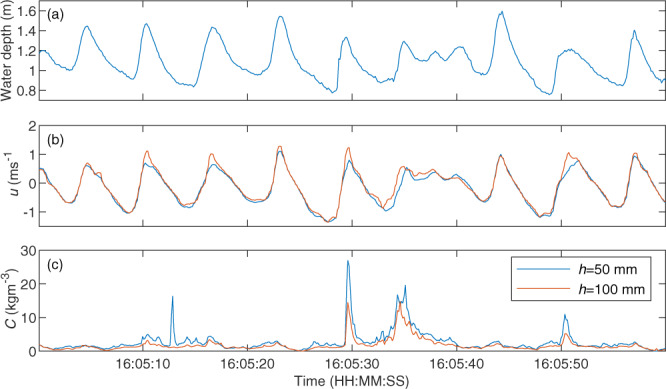
Timeseries data from surf zone rig 1, *x* = 226.5 m. (**a**) Water depth derived from pressure transducer data, **(b)** cross-shore flow velocity measured 5 cm (blue) and 10 cm (red) above the bed using EMCMs, and **(c)** suspended sediment concentrations 5 cm (blue) and 10 cm (red) above the bed measured using OBS.

Note that the two surf zone rigs described here were present for the entirety of Phase SB and the first 20 hours of the Phase DR testing. The instruments and scaffold rigs were removed during installation of the dynamic cobble berm revetment to avoid the risk of damage due to impact from stray cobbles from the revetment. Example post-processed data from the seaward surf zone rig is presented in Fig. 4.

### Swash zone measurements

The swash zone was monitored by a high definition IP camera (Vivotek IB9381-HT) which was used in RGB mode, the frame rate was 10 fps with a resolution of 2560 × 1920 px. The camera was mounted in the flume roof at *z* = 11.8 m landward of the runup limit, facing the wave paddle. The cross-shore position of the camera varied with the water level in the range *x* = 267 m to 280 m. A series of ground control points (GCPs) were positioned within the camera field of view to enable generation of rectified timestack images. The position of these GCPs was surveyed using the FARO Focus 3D terrestrial laser scanner.

**Fig. 5:**
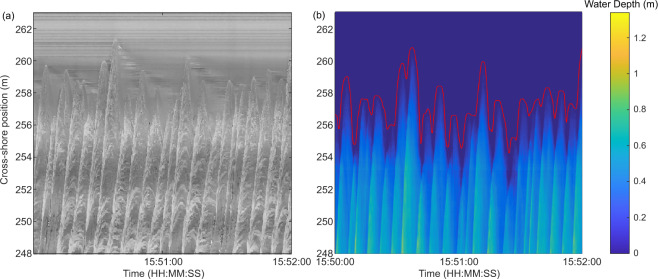
Example swash data. **(a)** Video timestack extracted from the high definition video. **(b)** Timestack of water depth extracted from the Lidar data with the timeseries of shoreline position added in red.

The timestack images of swash flow are complimented by the data from the most landward Lidar which monitored flow depths and bed elevations within the swash zone. Separation of the “bed” and “swash” using variance criteria^20^ as described above enables not only extraction of wave-by-wave bed elevations, but also estimates of the shoreline timeseries and depth-averaged flow velocity^21^ and capture of the bore collapse process^19^. Example swash zone measurements are presented in Fig. 5.

### Instrumented cobbles

The movement of individual cobbles within the dynamic revetment was monitored using an RFID tracking system similar to that previously used in field experiments^22^. The RFID system consists of three components: Passive Integrated Transponder (PIT) tags, the module reader and the antenna.

Texas Instruments TRPGR30ATGA PIT tags with a unique identification number and a detection range of 0.6 m were installed in 97 cobbles. The tags were placed inside 5 mm diameter holes drilled into the short axis of the cobbles and sealed using epoxy glue. Following PIT installation, the cobbles were washed, dried and painted in 3 different colours: 20 cobbles were painted pink and placed on the bottom layer of the revetment (at the sand interface) during its construction; 30 cobbles were painted orange and placed 20 cm above the bottom of the revetment (mid layer); 47 cobbles were painted green and placed at the toe and on the top layer of the revetment. All cobbles were placed along the centre line of the revetment in groups of 3 cobbles at 0.4 m cross-shore intervals. An additional 7 cobbles were initially placed at the revetment toe. Finally, the crest line of the revetment was painted yellow to enable modification of the crest by waves to be easily observed (Fig. 1c). Further details of the instrumented cobble placement are provided by Bayle *et al*.^6^ and the ‘DynaRev_RFID.xlsx’ spreadsheet provided in the dataset associated with this paper details the initial cobble positions and locations in each RFID survey.

The RFID reader used here was a Texas Instrument Series 2000 RI-STU-251B which transmits a radio frequency of 130.2 kHz and was connected to a logging computer via an RS232 serial connection. A 120 dB beeper was used to provide an audible beep when a PIT was detected. A Texas Instrument Ri-ANT-G02E antenna was connected to the module reader. The antenna measured 20 cm by 20 cm and was attached to a telescopic pole (up to 5 m long) to allow cobble detection from the side of the flume, avoiding the need for the operator to walk on, and potentially damage the revetment. Instrumented cobble surveys were completed at the end of each water level increment and day of testing during Phase DR by passing the antenna over the revetment surface in a systematic manner. The identification number and cross-shore position of each detected cobble was recorded for each survey.

## Data Records

The data detailed in this paper is available for download from 10.5281/zenodo.3889796^23^. Additional metadata is provided within each *.mat file detailing how the data from each instrument is stored. Note also that all raw, unprocessed data is available at 10.5281/zenodo.3860196^24^.

## Technical Validation

All data was collected using well-established coastal field and/or laboratory techniques using commercially available instrumentation. Post-processing was undertaken to remove outliers and convert spatial data to the *x, y, z* coordinate system defined above.

The profiler system provides the beach profile data directly in the local coordinate system (*x, z*). A visual check was completed directly after each profile to ensure no obvious measurement errors. Where errors were detected, the profile was repeated. The elevation data was interpolated onto a 0.025 m cross-shore grid.

The output from each Lidar provides the distance to the nearest target for every angle within each 2D scan at 25 Hz. This data was converted to local Cartesian coordinates (*x, z*) based on the position and orientation of each Lidar within the flume and interpolated onto a 0.1 m cross-shore grid. Outliers were only obtained where an object or person was positioned within the Lidar scan and these were removed manually. The exact location and orientation of the Lidar array was confirmed through comparison with the mechanical beach profiler data when no waves were running (see Fig. 3a). A RMSE smaller than 0.014 m was obtained.

Data from the wave gauges, ADVs, PTADV1 and PTADV2 (see Table 1) were sampled by the central GWK data acquisition system at 25 Hz. All wave gauges were calibrated at regular intervals throughout the experiment using a standard procedure. For each calibration, the water level was lowered from 5 m to 0.5 m in increments of 0.3 m and the voltage from all wave gauges at each water level was recorded for 180 s to create a calibration function relating water level to voltage. Wave gauge data was provided by the GWK system as a timeseries of water surface elevation in metres relative to the mean water level. ADV data was provided as *u, v, w* velocities (ms^−1^) and the pressure data were corrected for atmospheric pressure and provided in kPa.

In the surf zone, PTs were sampled at 8 Hz, corrected for atmospheric pressure and provided in kPa. EMCM data was sampled directly as *u, v* velocities at 8 Hz, no further post-processing was undertaken. The time-varying free surface elevations obtained from the Lidar data were compared with point measurements from pressure transducers PT1, PT2 and PT3 and wave gauge WGADV1 (see Table 1). For all runs the signals matched closely with zero lag.

All optical backscatter sensors were calibrated after the experiment to provide sediment concentration (gL^−1^) by applying the method of Betteridge *et al*.^25^ using sand from DynaRev in a specially constructed sediment tower at the University of Plymouth.

Camera timestacks were processed by extracting a line of pixels along the flume centreline and rectified using surveyed ground control points within the camera field of view.

## Data Availability

All code provided in DynaRev_Lib is written in MATLAB (R2019b). This folder contains the scripts used to process the raw data in order to obtain the post-processed data provided within the repository. The 3D Lidar point clouds described in Table 3 are provided in “.xyz” format which can be opened using the open source CloudCompare software package. The filename for each scan includes the date collected and the run after which the scan was completed, e.g. 20170918_DR2_7.xyz was completed after Run DR2–7 on 18^th^ September, 2017. A table providing the timings and notes about each scan is included within the DynaRev_3Dscans data record.

**Table 3:** Data files associated with the DynaRev experiment available from 10.5281/zenodo.3889796^23^.

Filename	Data description	Instruments (ref. Table 1)
DynaRev_TestProgram.xlsx	Complete list of test cases	—
DynaRev_Profiles.mat	Beach profiles measured after each run (*x,z*)	Mechanical profiler
DynaRev_Paddle_Files.zip	Wave paddle driver files in ascii format	Wave paddle
DynaRev_DAQ.mat	Timeseries data collected by the central data acquisition system: • Wave gauges - surface elevation, *η* (m) • ADVs – flow velocity, *u, v, w* (ms^−1^) • PTs – pressure, *P* (kPa) • Paddle stroke (m)	WG1 to 8, WGADV1 ADV1, ADV2, ADV3 PTADV2, PTADV3 Measured wave paddle stroke
DynaRev_SurfZone.mat	Timeseries data from the surf zone rigs: • PTs – pressure, *P* (kPa) • EMCMs – flow velocity, *u, v* (ms^−1^) • OBS – sediment concentration, *C* (gL^−1^)	PT1, PT2, PT3 OBS1 to OBS 4 EM1 to EM4
DynaRev_Lidar_<Phase><WL increment>-<Run No. >.mat	Timeseries x, z data from the combined Lidar array in.mat format. The data for each run is stored in a separate file, e.g. “DynaRev_Lidar_SB1–5.mat” contains the data for Phase SB, WL 1 (*z*_*wl*_ = 4.6 m), Run 1.	LID1, LID2, LID3
DynaRev_TimeStack.mat	Image timestack of swash zone	CAM
DynaRev_RFID.xlsx	Table containing instrumented cobble positions	RFID
DynaRev_3Dscans.zip	Point cloud data (*x,y,z* (m))from 11 3D Lidar scans of the morphology in “.xyz” format	FARO
DynaRev_Lib	Scripts for post-processing raw instrument data Data files associated with the DynaRev experiment available from 10.5281/zenodo.3889796^23^. Timeseries data collected by the central data acquisition system: • Wave gauges - surface elevation, *η* (m) • ADVs – flow velocity, *u, v, w* (ms^−1^) • PTs – pressure, *P* (kPa) • Paddle stroke (m) WG1 to 8, WGADV1 ADV1, ADV2, ADV3 PTADV2, PTADV3 Measured wave paddle stroke Timeseries data from the surf zone rigs: • PTs – pressure, *P* (kPa) • EMCMs – flow velocity, *u, v* (ms^−1^) • OBS – sediment concentration, *C* (gL^−1^) PT1, PT2, PT3 OBS1 to OBS 4 EM1 to EM4
