# Author Correction: High-resolution, large-scale laboratory measurements of a sandy beach and dynamic cobble berm revetment

**DOI:** 10.1038/s41597-021-00874-2

**Published:** 2021-03-15

**Authors:** Chris E. Blenkinsopp, Paul M. Bayle, Daniel C. Conley, Gerd Masselink, Emily Gulson, Isabel Kelly, Rafael Almar, Ian L. Turner, Tom E. Baldock, Tomas Beuzen, Robert T. McCall, Huub Rijper, Ad Reniers, Peter Troch, David Gallach-Sanchez, Alan J. Hunter, Oscar Bryan, Gwyn Hennessey, Peter Ganderton, Marion Tissier, Matthias Kudella, Stefan Schimmels

**Affiliations:** 1 Centre for Infrastructure, Geotechnics and Water Engineering, Department of Architecture and Civil Engineering, University of Bath, Bath, BA2 7AY UK; 2 School of Biological and Marine Sciences, Plymouth University, Drake Circus, PL4 8AA Plymouth, UK; 3 IRD-LEGOS, UMR5566, 18 Av. Edouard Belin, 31400 Toulouse, France; 4 Water Research Laboratory, School of Civil and Environmental Engineering, UNSW, Sydney, NSW 2052 Australia; 5 School of Civil Engineering, University of Queensland, Brisbane, QLD 4072 Australia; 6 Department of Statistics, University of British Columbia, Vancouver, Canada; 7 Department of Marine and Coastal Systems, Deltares, Boussinesqweg 1, 2629 HV Delft, the Netherlands; 8 Royal Boskalis Westminster N.V., Rosmolenweg 20, 3356 LK Papendrecht, the Netherlands; 9 Faculty of Civil Engineering and Geosciences, Delft University of Technology, Stevinweg 1, 2628 CN Delft, The Netherlands; 10 Department of Civil Engineering, Ghent University, Technologiepark 60, Ghent, B-9052 Belgium; 11 DEME Group, Scheldedijk 30, 2070 Zwijndrecht, Belgium; 12 Department of Mechanical Engineering, University of Bath, Bath, BA2 7AY UK; 13 Forschungszentrum Küste (FZK), Merkurstraße 11, 30419 Hannover, Germany

**Keywords:** Physical oceanography, Natural hazards

Correction to: *Scientific Data* 10.1038/s41597-021-00805-1, published online 20 January 2021

The original version of this Data Descriptor contained errors in the author affiliations. Peter Troch was incorrectly associated with DEME Group and the Department of Civil Engineering, Ghent University was inadvertently omitted. This has now been corrected in both the PDF and HTML versions of the Data Descriptor.

